# New perspectives in the treatment of chronic eosinophilic pneumonia in the era of targeted therapies

**DOI:** 10.3389/fimmu.2026.1893402

**Published:** 2026-08-14

**Authors:** Chiara Scelfo, Anna Simonazzi, Patrizia Ruggiero, Maria Rosaria Pellegrino, Nicola Facciolongo

**Affiliations:** Pulmonology Unit, Arcispedale Santa Maria Nuova, Azienda USL - IRCCS di Reggio Emilia, Reggio Emilia, Italy

**Keywords:** biologic therapies, chronic eosinophiiic pneumonia, eosino phils, IL5, steroid sparing drugs

## Abstract

**Background:**

Chronic eosinophilic pneumonia (CEP) is a rare, idiopathic inflammatory lung disease characterized by subacute symptoms such as cough, dyspnea, and fever; abnormal chest imaging findings; peripheral eosinophilia; and eosinophilic infiltration of the pulmonary interstitium and alveoli. Eosinophils interact with multiple T helper 2 (Th2) cellular pathways; however, eosinophil recruitment to the lung is primarily mediated by interleukin (IL)-5 and the eotaxin subfamily of chemokines. Standard treatment consists of systemic corticosteroids; however, relapse is frequent after tapering the corticosteroid dose, leading to long-term steroid therapy and its associated adverse effects, including diabetes mellitus, infections, glaucoma, and osteoporosis.

**Objective:**

This narrative review aims to summarize the available evidence on the effectiveness and safety of anti–IL-5 biologic therapies (mepolizumab, reslizumab, and benralizumab) in the management of CEP and as steroid-sparing strategies, with particular focus on their role in relapse prevention, which occurs in nearly 50% of cases during systemic corticosteroid tapering.

**Methods:**

A narrative review was conducted to identify the real-world advances of anti-IL-5 treatments in CEP. Most of the available literature consists of case series, case reports and observational studies, owing to the lack of controlled clinical trials and guidelines involving this disease and anti–IL-5 therapies.

**Results:**

The available evidence suggests that anti–IL-5 therapies are effective in controlling disease activity, reducing relapse rates, and enabling corticosteroid tapering or discontinuation.

**Conclusions:**

All the treatments considered were safe and well tolerated, and their use provided significant benefits in terms of symptom control, despite the limited impact on pulmonary function. Owing to their steroid-sparing effects, these agents are useful in reducing the adverse effects associated with long-term corticosteroid use. Anti–IL-5 biologics represent a promising therapeutic option for patients with relapsing or corticosteroid-dependent CEP. Despite encouraging results, larger prospective studies are needed to better define their role in the long-term management of this disease.

## Introduction

Chronic eosinophilic pneumonia (CEP) is a rare idiopathic inflammatory disorder characterized by accumulation of eosinophils in the lung and, frequently, peripheral blood eosinophilia, with unknown etiology, that usually occurs in middle-aged women with female sex predominance (2:1), diagnosed by clinical and laboratory findings associated with abnormal chest imaging, characterized by multiple foci of consolidation in peripheral lung fields. Eosinophils play a central role in the pathogenesis of the disease, and their pathophysiological mechanisms have been extensively investigated and are well documented in the literature. The etiology is heterogeneous, encompassing fungal and parasitic infections, drugs, toxins, autoimmune inflammatory disorders, and neoplasms. In cases where no underlying cause is identified, the condition is considered primary or idiopathic, representing a diagnosis of exclusion.

Clinically the presentation is characterized by malaise, fever, night sweats, anorexia, and weight loss, cough and dyspnea. It is strong associated with asthma (50%) with the severity of asthma increasing after the onset of CEP, allergic rhinitis and atopy, including eczema, urticaria and nasal polyposis (1). Airway remodeling caused by the chronic accumulation of eosinophilic inflammation leads to a progressive decline in pulmonary function (2), which may be further worsened by the development of significant and irreversible fibrosis. Diagnosis is based on clinical evaluation, radiological findings at high-resolution computed tomography (HRCT) characterized by fluffy pulmonary opacities, and the exclusion of alternative etiologies, including parasitic, fungal, iatrogenic, vasculitic, and granulomatous diseases. Nevertheless, bronchoalveolar lavage fluid (BALF) analysis demonstrating eosinophilia remains a key diagnostic tool (3). In addition, increased and heterogeneous levels of type 2 cytokines have been detected in the bronchoalveolar lavage fluid (BALF) of these patients and are believed to contribute to eosinophil activation and accumulation. Careful diagnostic evaluation is therefore essential, as eosinophilic pneumonia may also represent a manifestation of a more severe systemic disorder within the spectrum of vasculitic diseases, such as eosinophilic granulomatosis with polyangiitis (EGPA), which requires a distinct diagnostic and therapeutic approach, often including immunosuppressive agents. Systemic corticosteroids remain the cornerstone of treatment and generally lead to rapid clinical and radiological improvement. Early recognition is crucial to prevent progression to respiratory failure and to avoid unnecessary antimicrobial therapy. Despite these favorable outcomes, important clinical challenges persist in patients with CEP, particularly the high relapse rate observed during corticosteroid tapering or after treatment discontinuation, as well as the lack of evidence-based recommendations regarding the optimal dosage and duration of therapy.

The frequent occurrence of relapses often necessitates prolonged or maintenance corticosteroid treatment, exposing patients to potentially severe adverse effects, including osteoporosis, diabetes mellitus, cataracts, impaired wound healing, and increased susceptibility to infections. These limitations have prompted growing interest in steroid-sparing therapeutic strategies. In this context, the recent development of biologic agents has opened new therapeutic perspectives, particularly with monoclonal antibodies targeting interleukin-5 (IL-5), for the management of refractory eosinophilic pneumonia and in patients who are intolerant to oral corticosteroids (OCS).

This narrative review aims to evaluate the current evidence regarding alternative therapies to OCS in eosinophilic pneumonia. Particular attention is given to the most recent data, which are mainly derived from case reports and case series, in order to explore the efficacy, safety, and potential clinical role of steroid-sparing therapeutic approaches.

## Physiological roles of eosinophils

**Figure 1:**
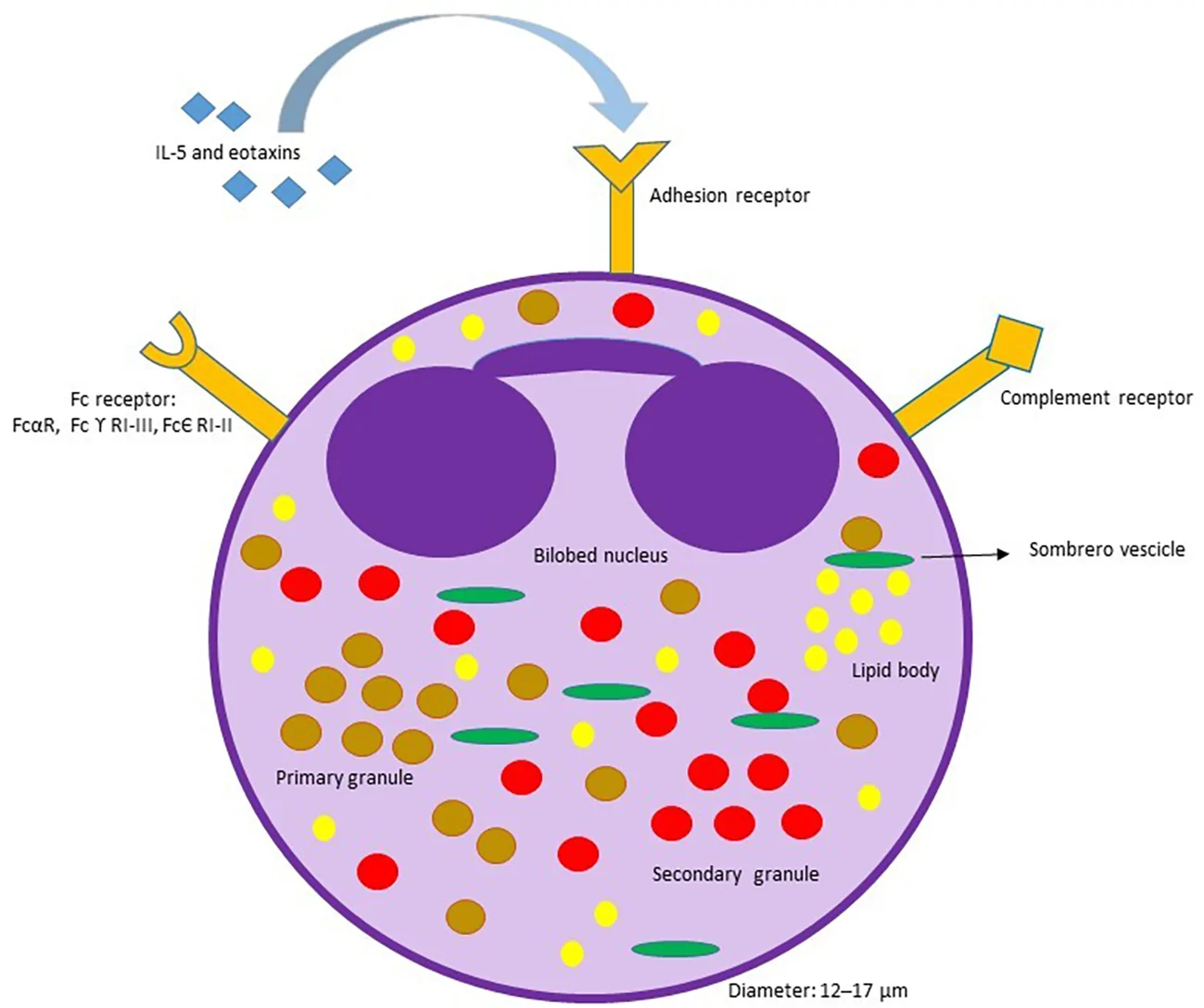
Eosinophil.

Eosinophils are a specialized subset of granulocytes, typically identified across species by their distinctive red or pink cytoplasmic granules, which reflect the strong affinity of their contents for eosin dye in microscopy (**Figure 1**). They represent an evolutionarily conserved cell type, being present across all vertebrate lineages; however, despite their long evolutionary history, their precise biological functions remain only partially understood (4–6).

A defining feature of eosinophils is the presence of specific secondary granules containing a wide range of preformed mediators capable of inducing inflammation and tissue damage, distinguishing them from other granulocytes. In humans, eosinophils are relatively rare, accounting for approximately 1–3% of circulating leukocytes, corresponding to about 100 cells/μL. Normal blood eosinophil levels are generally considered to be below 500 cells/μL; eosinophilia is defined as a count exceeding this threshold, while hypereosinophilia refers to levels greater than 1,500 cells/μL (7).

In response to specific stimuli, eosinophils may survive for several weeks, challenging the traditional concept of these cells as short-lived leukocytes. Increasing evidence suggests that eosinophils represent a heterogeneous cell population that can be classified into distinct phenotypic and functional subsets, reflecting their diverse roles in tissue homeostasis and immune regulation. Proposed subsets include eosinophil progenitors, short-lived circulating eosinophils, steady-state or tissue-resident eosinophils, and the functionally distinct E1 and E2 phenotypes. Tissue-resident eosinophils are thought to play a key role in maintaining homeostasis in organs such as the lungs, adipose tissue, and gastrointestinal tract, whereas the E1 and E2 subsets are primarily associated with inflammatory responses, particularly within interstitial and epithelial compartments (8–10) (**Table 1**).

**Table 1:** Phenotypic and functional heterogeneity of eosinophils in homeostasis and disease.

Eosinophil subset	Primary location	Phenotypic markers	Main functional characteristics	Clinical relevance
Tissue-resident eosinophils (rEos)	Healthy lung parenchyma, gastrointestinal tract, thymus, adipose tissue, uterus	Siglec-8+, IL-5Rα+, CD62L^high^, CD101^low^ (murine counterpart: Siglec-F^int^, CD62L+, CD101−)	Maintain tissue homeostasis, support epithelial integrity, regulate local immune responses, promote tissue repair and metabolic homeostasis	Homeostatic population largely independent of active inflammation
Inflammatory eosinophils (iEos)	Inflamed airways, lung, nasal polyps, skin, gastrointestinal tract	Activated phenotype; CD101^high^, CD11b^high^, CD69+, IL-5Rα+, CCR3+, reduced CD62L (murine counterpart: Siglec-F^high^)	Release cytotoxic granule proteins, cytokines, lipid mediators and extracellular traps; amplify type 2 inflammation	Major effector population in asthma, CEP, EGPA, CRSwNP and eosinophilic gastrointestinal diseases
Activated eosinophils	Peripheral blood and inflamed tissues	CD69+, CD44+, CD11b+, CD63+, CD66b+, decreased CD62L	Enhanced adhesion, migration, degranulation and prolonged survival	Correlate with disease activity and acute eosinophilic inflammation
Hypodense eosinophils	Peripheral blood during severe eosinophilic disorders	Reduced density after gradient centrifugation; activated phenotype	Increased cytokine production, prolonged survival, enhanced degranulation	Frequently detected in severe asthma, HES and EGPA; associated with active disease
Normodense eosinophils	Peripheral blood under physiological conditions	Resting phenotype	Immune surveillance and precursor pool for tissue recruitment	Predominant circulating population in healthy individuals
Regulatory/ homeostatic eosinophils	Gastrointestinal tract, adipose tissue, secondary lymphoid organs	Variable phenotype; expression of immunomodulatory mediators	Promote IgA-producing plasma cell survival, regulate T-cell responses, support macrophage polarization and tissue remodeling	Contribute to immune tolerance and maintenance of tissue homeostasis
Pathogenic type 2 eosinophils	Lung and airways during type 2 inflammation	IL-5Rα+, CCR3+, Siglec-8+, activated IL-5 signaling	Produce IL-4, IL-5, IL-13, TGF-β, eosinophil cationic proteins and extracellular traps	Central drivers of eosinophilic asthma, CEP and other IL-5-dependent diseases; principal target of anti-IL-5/IL-5R biologics
ANCA-associated eosinophils (EGPA phenotype)	Blood and tissues affected by vasculitis	Activated phenotype with increased adhesion molecules and tissue infiltration	Interact with neutrophils, endothelial cells and adaptive immune cells; contribute to vascular injury and granuloma formation	Characteristic of eosinophilic granulomatosis with polyangiitis, particularly during active disease

Much of the current understanding of eosinophil function in physiological homeostasis and health is derived from animal studies, particularly murine models. In this context, eosinophils are increasingly recognized as multifunctional cells involved in tissue development, remodeling, and repair through the release of a broad array of bioactive mediators, especially at barrier sites such as epithelial surfaces. Beyond these structural and reparative roles, eosinophils participate in both innate and adaptive immune responses by modulating immune cell development and function and by contributing to host defense against parasitic, bacterial, fungal, and viral pathogens (**Table 1**).

In addition, accumulating evidence indicates that eosinophils are involved in the regulation of several systemic physiological processes, including glucose metabolism, skeletal muscle regeneration, brown adipose tissue development and adiposity, liver repair, and the maintenance of neuronal function and microbiome homeostasis (**Table 1**). Collectively, eosinophils also exert important immunoregulatory functions that may contribute to the preservation of immunologic fitness during aging (7, 11–13).

### Eosinophil differentiation

Eosinophils originate in the bone marrow from multipotent hematopoietic stem cells. Commitment to the myeloid lineage gives rise to myeloblasts with shared characteristics of basophils and eosinophils, which subsequently differentiate into a distinct eosinophil lineage. This process is tightly regulated by a network of transcription factors—including C/EBP, PU.1, GATA-1, Helios, Aiolos, and XBP1—as well as soluble mediators such as interleukin (IL)-3, IL-5, and granulocyte–macrophage colony-stimulating factor (GM-CSF), ultimately leading to the generation of mature eosinophils (7, 14).

A key step in eosinophil lineage commitment is the expression of the IL-5 receptor on the cell surface. This heterodimeric receptor, which can also interact with IL-3 and GM-CSF, represents one of the earliest eosinophil-specific markers detectable in the bone marrow. While IL-5 has long been considered the principal driver of eosinophil differentiation, emerging evidence indicates that IL-33 may act upstream, enhancing eosinophil development prior to IL-5–mediated effects (15).

Mature eosinophils are terminally differentiated granulocytes that have lost their proliferative capacity and are released from the bone marrow into the bloodstream following changes in their adhesive and migratory properties. In humans, eosinophils have a relatively short circulating lifespan of approximately 8–18 hours before migrating into peripheral tissues. Under physiological conditions, they are predominantly tissue-resident cells, with the gastrointestinal tract, extending from the stomach to the intestine, representing their principal site of localization, where they contribute to tissue homeostasis and immune regulation.

The recruitment of eosinophils from the circulation into tissues is orchestrated by a coordinated interaction between adhesion molecules and chemotactic signals. Adhesion is primarily mediated by β1- and β2-integrins expressed on eosinophils and their endothelial ligands, including vascular cell adhesion molecule-1 (VCAM-1), whereas directed migration is driven by eosinophil-selective chemokines, particularly the eotaxin family (CCL11, CCL24, and CCL26), acting through the chemokine receptor CCR3. Once within tissues, eosinophils typically survive for several days under homeostatic conditions. However, during type 2 inflammatory responses, their lifespan is markedly prolonged by pro-survival cytokines, including interleukin-5 (IL-5), granulocyte–macrophage colony-stimulating factor (GM-CSF), and interleukin-3 (IL-3), which inhibit apoptosis and promote eosinophil activation, persistence, and effector functions. As a result, tissue eosinophils may survive for several weeks, thereby sustaining chronic eosinophilic inflammation (16, 17).

### Eosinophil activation

Mature eosinophils contain characteristic cytoplasmic granules with a crystalloid core primarily composed of major basic proteins (MBP-1 and MBP-2), surrounded by a matrix rich in eosinophil cationic protein (ECP), eosinophil-derived neurotoxin (EDN), and eosinophil peroxidase (EPO), as well as cytosolic Charcot–Leyden crystal protein/galectin-10 (CLC/Gal-10). These granule-associated proteins, particularly MBP, EPO, and ECP, are potent mediators of tissue injury and dysfunction, contributing to damage in multiple organs, including the heart, central nervous system, and bronchial epithelium (14).

Upon activation, eosinophils can release their granular contents through three principal mechanisms. Classical exocytosis involves the fusion of specific granules with the plasma membrane. The most common pathway is piecemeal degranulation, in which selected granule components are selectively transported and secreted via cytoplasmic vesicles or membrane-associated processes. In contrast, cytolytic degranulation is characterized by cell membrane disruption, resulting in the extracellular release of intact, cell-free granules and CLC protein, along with the formation of eosinophil extracellular traps (EETs), which consist of networks of nuclear DNA fibers (8, 18, 19).

### Interleukin-5: a key cytokine in type 2 inflammation

Interleukin-5 (IL-5) is a central mediator of eosinophilic inflammation. Targeted biologic therapies directed against IL-5 or its receptor (IL-5R) have demonstrated efficacy in reducing eosinophil counts and improving clinical outcomes in diseases driven by type 2 (Th2-mediated) immune responses (20).

*In vivo*, IL-5 is produced by multiple cell types, including T helper 2 (Th2) cells, innate lymphoid cells (ILCs), mast cells, basophils, and dendritic cells. Evidence from ex vivo studies, experimental models, and clinical interventions indicates that IL-5 exerts pleiotropic effects on a wide range of cell populations beyond eosinophils, including epithelial cells, plasma cells, mast cells, basophils, neutrophils, group 2 innate lymphoid cells (ILC2s), regulatory T (Treg) cells, and fibroblasts (21–23).

Despite these broad effects, eosinophils remain the primary effector cells responsive to IL-5 signaling. The biological actions of IL-5 on eosinophils can be broadly categorized into four key processes: differentiation, migration, activation, and survival. IL-5 promotes the mobilization of eosinophils from the bone marrow to the bloodstream and subsequently to peripheral tissues, in part through β2 integrin–mediated adhesion mechanisms, including the CD11b/CD18 (Mac-1) complex, thereby enhancing eosinophil adherence to endothelial cells (24, 25).

In the peripheral circulation, IL-5 supports the terminal differentiation of eosinophil precursors and prolongs eosinophil survival by inhibiting apoptosis, as demonstrated in both human and murine studies. This results in increased eosinophil numbers in blood and tissues (26). During allergen-driven adaptive immune responses, IL-5 is predominantly produced by Th2 cells, whereas in innate immune settings, ILC2s represent a major source of IL-5, particularly in response to epithelial-derived alarmins such as IL-33 (27).

At the molecular level, IL-5 signaling in mature eosinophils activates multiple intracellular pathways, including the Janus kinase–signal transducer and activator of transcription (JAK–STAT) and mitogen-activated protein kinase (MAPK) pathways, which are critical for eosinophil proliferation, survival, and effector function. IL-5 also regulates the expression of genes involved in eosinophil and B-cell lineage development, maturation, and functional activity (28, 29).

### Mechanisms for the development of eosinophilic airway accumulation and activation

Eosinophils accumulate at sites of inflammation and play a central role in disease pathogenesis through the release of a wide range of mediators, including granule-derived proteins, reactive oxygen species, and cytokines. IL-5, a hematopoietic growth factor belonging to the cytokine family, is a key regulator of eosinophil priming and survival (30).

**Figure 2:**
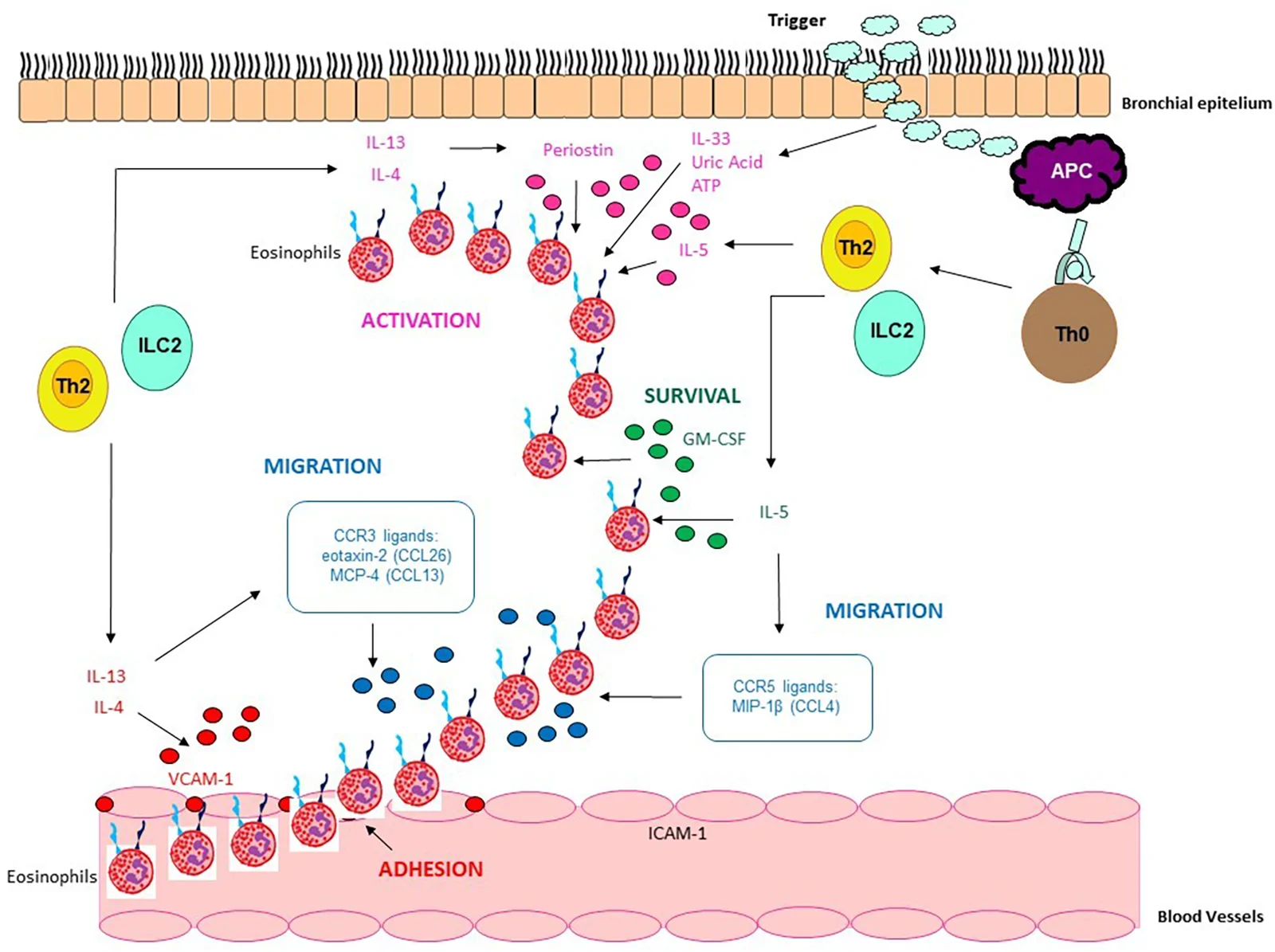
Potential mechanism involved in eosinophil accumulation in the airways in eosinophilic pneumonia.

Eosinophil migration and adhesion to the endothelium are primarily regulated by type 2 cytokines, such as IL-4 and IL-13, as well as chemokines associated with Th2 cells and ILC2s. These processes involve interactions between α4 integrins and vascular cell adhesion molecule-1 (VCAM-1), along with the activation of chemokine receptors, particularly CCR3 (**Figure 2**). CCR3 is highly and constitutively expressed on eosinophils, and its ligands—including eotaxin (CCL11), eotaxin-2 (CCL24), eotaxin-3 (CCL26), RANTES (CCL5), and MCP-4 (CCL13)—act as potent chemoattractants during allergic inflammation. Notably, these ligands are overexpressed in the airways of patients with asthma and eosinophilic pneumonia.

In eosinophilic pneumonia, increased concentrations of eotaxin, eotaxin-2, and MCP-4 have been detected in BALF, contributing to eosinophil recruitment and transendothelial migration. Experimental evidence indicates that blockade of CCR3 or its ligands significantly reduces eosinophil migration, underscoring the pivotal role of the CCR3–ligand axis in disease pathogenesis (30–32). Additional chemokines, including CCR5 ligands such as macrophage inflammatory protein-1β (MIP-1β), also contribute to eosinophil trafficking (33).

Following transmigration into tissues, eosinophil activation and degranulation are driven by IL-5, IL-3, and GM-CSF, as well as lipid mediators such as cysteinyl leukotrienes (cysLTs) (31, 34). Other molecules, including extracellular matrix proteins such as periostin, have also been implicated in eosinophil accumulation and activation in eosinophilic pneumonia (**Figure 2**) (35). In this context, biologic therapies targeting key cytokines involved in these pathways—particularly IL-5—represent a promising therapeutic strategy.

## Differential diagnosis of chronic eosinophilic pneumonia

The diagnosis of CEP requires the exclusion of several eosinophilic lung diseases, including EGPA, allergic bronchopulmonary aspergillosis (ABPA), drug-induced eosinophilic pneumonia, parasitic infections, hypereosinophilic syndrome (HES), and eosinophilic malignancies. Among these conditions, EGPA represents the most challenging differential diagnosis because both disorders share peripheral blood eosinophilia, pulmonary infiltrates, asthma, and a marked response to systemic corticosteroids. Consequently, a comprehensive clinical assessment integrating radiological, laboratory, and extrapulmonary findings is essential.

### Distinguishing CEP from eosinophilic granulomatosis with polyangiitis

EGPA is a systemic eosinophilic vasculitis affecting small- and medium-sized vessels. Although pulmonary infiltrates are common, they are usually accompanied by extrapulmonary manifestations such as mononeuritis multiplex, purpura, sinonasal disease, cardiomyopathy, gastrointestinal involvement, or renal disease. Histologically, EGPA is characterized by necrotizing vasculitis, eosinophil-rich granulomatous inflammation, and tissue eosinophilia. Antineutrophil cytoplasmic antibodies (ANCA), particularly myeloperoxidase (MPO)-ANCA, are detected in approximately 30–40% of patients and, although not diagnostic, strongly support the diagnosis when associated with compatible clinical features (8, 36).

Several clinical clues may help distinguish CEP from EGPA (**Table 2**). Asthma in CEP frequently develops shortly before or concomitantly with pulmonary disease and is often the predominant manifestation, whereas EGPA usually follows a characteristic sequence of severe adult-onset asthma, chronic rhinosinusitis with nasal polyposis, eosinophilic tissue infiltration, and eventually systemic vasculitis. Peripheral neuropathy, palpable purpura, constitutional symptoms, and cardiac involvement strongly favor EGPA, whereas isolated pulmonary disease with characteristic peripheral consolidations and absence of systemic manifestations is more suggestive of CEP. Importantly, recurrent or corticosteroid-dependent CEP should prompt careful long-term follow-up, as rare cases initially diagnosed as CEP have subsequently evolved into overt EGPA, suggesting that these disorders may occasionally represent different phenotypic expressions within the spectrum of eosinophilic diseases (1).

**Table 2:** Differential diagnosis between chronic eosinophilic pneumonia (CEP) and eosinophilic granulomatosis with polyangiitis (EGPA).

Feature	Chronic eosinophilic pneumonia (CEP)	Eosinophilic granulomatosis with polyangiitis (EGPA)
Disease type	Idiopathic eosinophilic lung disease	ANCA-associated necrotizing vasculitis
Typical age	30–60 years	40–60 years
8Asthma	Present in 60–90%; often precedes or accompanies pulmonary disease	Present in >90%; usually severe, adult-onset, longstanding
Blood eosinophilia	Usually >1,000 cells/μL	Usually >1,000 cells/μL; often marked
BAL eosinophils	Frequently >40%	Often elevated but variable
Chest HRCT	Bilateral peripheral consolidations, upper lobe predominance; ground-glass opacities	Transient or migratory consolidations, ground-glass opacities, nodules, bronchial wall thickening; less characteristic distribution
Upper airway disease	Occasionally rhinitis	Chronic rhinosinusitis and nasal polyposis are common
Peripheral neuropathy	Absent	Common (especially mononeuritis multiplex)
Skin manifestations	Rare	Purpura, nodules, urticaria frequently observed
Cardiac involvement	Exceptional	One of the major causes of mortality
Renal involvement	Absent	Present in a subset of patients, particularly ANCA-positive
ANCA	Usually negative	Positive in ~30–40% (predominantly MPO-ANCA)
Histopathology	Alveolar/interstitial eosinophilic infiltrates without vasculitis	Eosinophilic infiltrates, necrotizing vasculitis, extravascular granulomas
Response to corticosteroids	Rapid, often dramatic	Usually good, although relapses are common
Relapse pattern	Frequent during steroid tapering	Depends on vasculitic activity and eosinophilic inflammation
Biologic therapy	Increasing evidence for anti-IL-5/IL-5R biologics	Anti-IL-5 biologics recommended in relapsing or refractory disease

The introduction of the 2022 American College of Rheumatology/European Alliance of Associations for Rheumatology (ACR/EULAR) classification criteria has improved the standardization of EGPA classification in research settings. These criteria assign weighted scores to peripheral eosinophilia (≥1×10^9^/L), obstructive airway disease, nasal polyposis, mononeuritis multiplex, and histological evidence of eosinophilic inflammation, whereas PR3-ANCA positivity and hematuria decrease the cumulative score. A total score of ≥6 classifies patients as EGPA after alternative diagnoses have been excluded. However, these criteria are intended for disease classification rather than diagnosis and should not replace clinical judgment, particularly in patients presenting with isolated pulmonary eosinophilic disease (37).

ANCA testing may further assist the differential diagnosis but has important limitations. Approximately 30–40% of patients with EGPA are MPO-ANCA positive, whereas CEP is typically ANCA-negative. Nevertheless, the majority of eosinophilic-predominant EGPA cases are also ANCA-negative, indicating that a negative ANCA result cannot reliably exclude vasculitis. Similarly, elevated serum IgE, thymus and activation-regulated chemokine (TARC/CCL17), eosinophil-derived neurotoxin (EDN), or eosinophil cationic protein (ECP) reflect eosinophilic inflammation but currently lack sufficient specificity for routine differential diagnosis (8, 36).

An emerging concept is that CEP and EGPA may not always represent completely distinct diseases but rather different phenotypic manifestations along a spectrum of eosinophilic disorders driven by type 2 inflammation. Several longitudinal studies have documented patients initially diagnosed with CEP who subsequently developed systemic vasculitic manifestations fulfilling criteria for EGPA months or years later (38). Although this evolution appears uncommon, these observations emphasize the importance of long-term follow-up in patients with recurrent, corticosteroid-dependent, or atypical CEP, particularly when eosinophilia persists despite adequate therapy or extrapulmonary symptoms emerge.

Overall, distinguishing CEP from EGPA requires an integrated multidisciplinary approach combining clinical assessment, imaging, bronchoscopy, laboratory investigations, ANCA testing, and, whenever indicated, tissue biopsy. Rather than relying on a single diagnostic test, clinicians should recognize the dynamic nature of eosinophilic diseases and maintain vigilance for the subsequent development of systemic vasculitis during longitudinal follow-up.

### Treatment of CEP: standard therapies and IL-5/IL-5 receptor-targeted therapies

CEP typically responds well to systemic corticosteroid therapy and, in a minority of cases (<10%), may resolve spontaneously. Clinical remission is usually achieved within approximately two weeks with an initial dose of around 0.5 mg/kg of prednisolone; however, relapse rates remain high. In the absence of standardized treatment protocols, corticosteroids are generally tapered gradually over a prolonged period—often extending from six months to one year—resulting in substantial cumulative exposure.

Shorter treatment courses have been associated with increased relapse rates, as demonstrated by Oyama et al., who reported no significant difference in relapse frequency between 3- and 6-month regimens (39). Recurrent disease further increases cumulative corticosteroid exposure, raising the risk of long-term adverse effects. Alternative approaches, such as inhaled corticosteroids, have not demonstrated significant clinical benefit or reduction in relapse rates, as shown in a study by Minakuchi et al. evaluating budesonide (40).

Given the central role of IL-5 in eosinophilic inflammation and the availability of targeted biologic therapies for severe asthma, these agents have increasingly been considered for the management of CEP. Their use has been explored not only in refractory cases but also as a potential steroid-sparing strategy in patients at high risk of corticosteroid-related complications or in those with frequent relapses (41).

**Figure 3:**
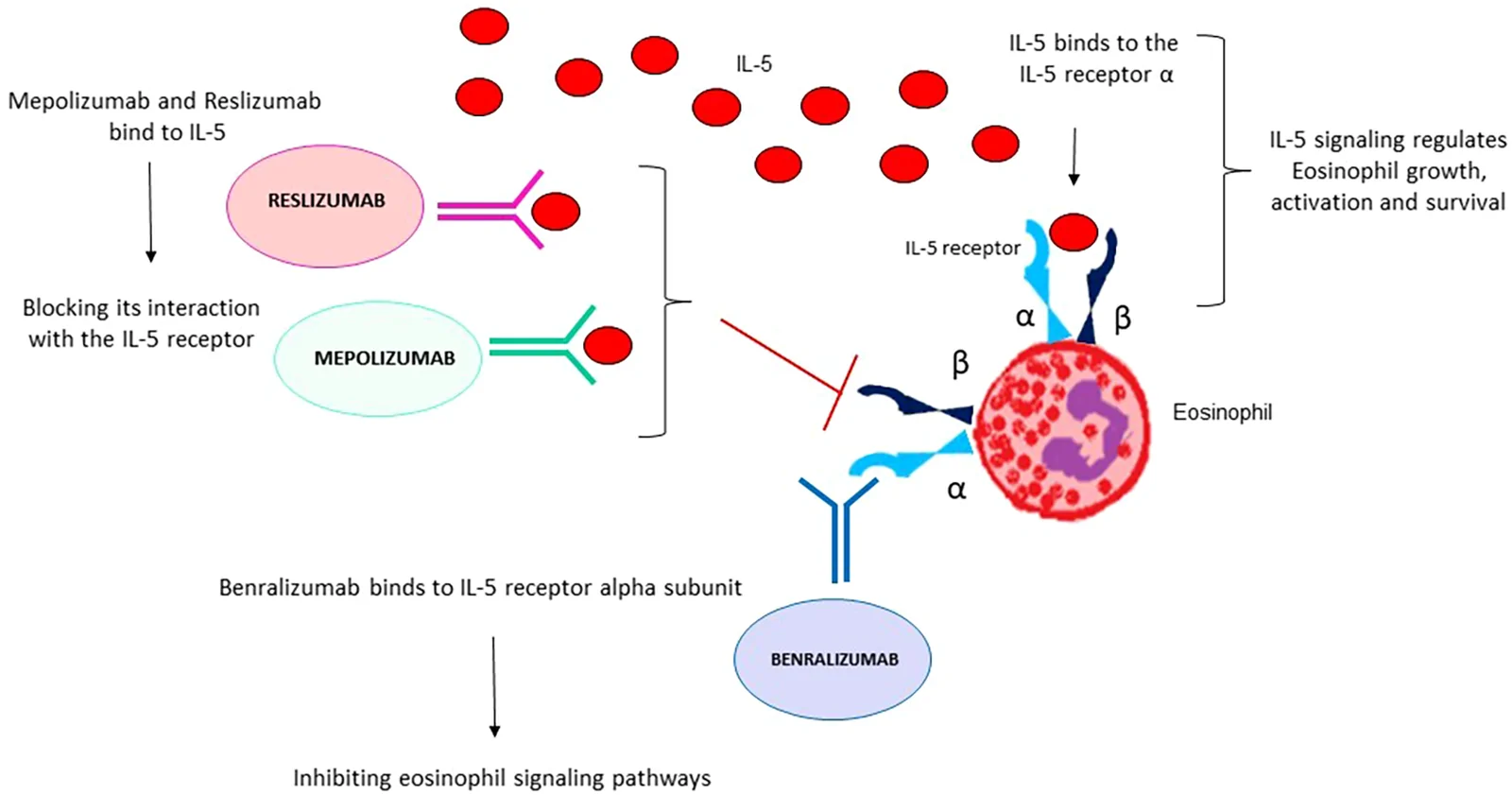
Mechanisms of action of anti–IL-5 biologic therapies and their effects on eosinophil maturation and differentiation. Mepolizumab and reslizumab neutralize circulating IL-5, preventing its interaction with the IL-5 receptor and thereby inhibiting eosinophil maturation and survival. Benralizumab targets the IL-5 receptor α (IL-5Rα) on eosinophils and basophils, inducing antibody-dependent cell-mediated cytotoxicity and leading to rapid eosinophil depletion.

Mepolizumab and Reslizumab are monoclonal antibodies directed against IL-5, preventing its interaction with the IL-5 receptor α (IL-5Rα) on eosinophils and thereby reducing eosinophil maturation, activation, and survival (**Table 3**). In contrast, Benralizumab targets the IL-5Rα expressed on eosinophils and basophils, inducing antibody-dependent cell-mediated cytotoxicity and leading to rapid and near-complete eosinophil depletion (**Figure 3**). Benralizumab has attracted particular interest because its mechanism of direct eosinophil depletion appears to induce faster and more profound suppression of eosinophilic inflammation than IL-5 neutralization alone. Recent reports suggest that benralizumab may maintain prolonged remission even with extended dosing intervals, raising the possibility of individualized treatment schedules based on eosinophil kinetics (42).

**Table 3:** Overview of selected eosinophil-specific therapies.

TARGET	DRUG	Administration	Dose	Primary indications	Advantages
IL-5	MEPOLIZUMAB	Subcoutaneous	100 mg every 4 weeks	Severe eosinophilic asthma, CRSWNP	Well-established safety profile and durable therapeutic effect
			300 mg every 4 weeks	HES, EGPA,	
	RESLIZUMAB	Intravenous	3 mg kg-1 every 4 weeks	Severe eosinophilic asthma	Personalized, weight-adjusted dosing suitable for overweight patients
IL-5 Ra	BENRALIZUMAB	Subcoutaneous	30 mg every 8 weeks	Severe eosinophilic asthma	High-affinity binding to the IL-5 receptor with ADCC-mediated near-complete eosinophil depletion, administered every 8

These biologic therapies have been widely used in severe eosinophilic asthma, where they have demonstrated favorable safety profiles and substantial clinical efficacy, including the ability to reduce or discontinue systemic corticosteroid therapy.

Another biologic treatment that has recently attracted considerable interest is depemokimab, following its approval for the treatment of severe eosinophilic asthma. Its unique pharmacokinetic profile and prolonged duration of action distinguish it from currently available anti-IL-5 therapies, making it a promising candidate for the long-term management of eosinophilic disorders.

Depemokimab is a novel, ultra-long-acting humanized monoclonal antibody targeting interleukin-5 (IL-5), specifically engineered to provide sustained suppression of eosinophilic inflammation with a dosing interval of up to six months. By combining high-affinity IL-5 neutralization with an extended half-life, depemokimab represents the next generation of anti-IL-5 biologics and has recently demonstrated significant efficacy in reducing exacerbations in patients with severe eosinophilic asthma (43).

Although depemokimab has not yet been evaluated in chronic eosinophilic pneumonia, its prolonged half-life and sustained IL-5 neutralization, allowing twice-yearly administration, make it an attractive candidate for future investigation in patients with relapsing or corticosteroid-dependent CEP. Its extended dosing interval may improve treatment adherence while further reducing cumulative corticosteroid exposure, although dedicated clinical studies are required before its use can be recommended in this setting.

## From phenotype to therapy: selecting the optimal anti-IL-5 biologic in chronic eosinophilic pneumonia

At present, no evidence-based recommendations or head-to-head comparative studies are available to guide the selection of mepolizumab, reslizumab, or benralizumab in patients with CEP. Consequently, treatment decisions are largely individualized and are generally extrapolated from clinical experience in severe eosinophilic asthma and EGPA. In routine clinical practice, several patient-related factors may influence biologic selection, although none has been prospectively validated specifically for CEP.

Baseline blood eosinophil count is often considered when initiating biologic therapy. While all three agents are effective in eosinophilic diseases, benralizumab induces nearly complete depletion of circulating and tissue eosinophils through antibody-dependent cell-mediated cytotoxicity targeting IL-5 receptor α (IL-5Rα). Consequently, some clinicians preferentially select benralizumab in patients with marked hypereosinophilia, recurrent relapses despite corticosteroid therapy, or when rapid suppression of eosinophilic inflammation is considered desirable. In contrast, mepolizumab, which directly neutralizes IL-5, has accumulated the largest body of published evidence in CEP, including the greatest number of case reports and case series, making it the biologic with the strongest real-world experience in this setting despite the absence of randomized controlled trials. Reslizumab has been reported only sporadically in CEP and is therefore generally reserved for selected patients or when alternative agents are unavailable. The presence of comorbid eosinophilic disorders also plays an important role in therapeutic decision-making. In patients with severe eosinophilic asthma requiring biologic therapy, clinicians frequently select the agent expected to provide optimal control of both diseases. Mepolizumab is often favored in patients with concomitant EGPA because of its regulatory approval and demonstrated efficacy in reducing disease relapses and oral corticosteroid exposure. Conversely, benralizumab may be preferred in individuals with severe eosinophilic asthma characterized by persistent blood eosinophilia, frequent exacerbations, or inadequate response to previous anti-IL-5 therapy, owing to its more profound eosinophil depletion. Similarly, the coexistence of chronic rhinosinusitis with nasal polyps may influence treatment selection, as both mepolizumab and benralizumab have demonstrated efficacy in this condition. Practical considerations may also affect biologic choice in routine care. Benralizumab is administered every four weeks for the first three doses and every eight weeks thereafter, whereas mepolizumab requires monthly administration throughout treatment.

## Discussion

The use of anti–IL-5 therapies is not currently supported by established guidelines or standardized treatment protocols; however, several case reports and small case series have described their effective use, particularly in patients experiencing disease relapse or in those for whom long-term OCS therapy is not feasible due to adverse effects or intolerance (44–47). In a retrospective study including 10 patients with CEP, treatment with mepolizumab reduced the annual relapse rate to zero and enabled prednisone tapering in all cases, with near-complete resolution of lung infiltrates on follow-up imaging in the majority of patients (45). Importantly, this study suggested that the early introduction of biologic therapy might prevent progression toward pulmonary fibrosis, although this hypothesis requires confirmation in larger prospective studies.

A recent scoping review reported more than 60 patients with CEP treated with anti–IL-5 agents; nearly all experienced clinical improvement, with an absence of further relapses and a substantial reduction in maintenance corticosteroid doses (44). Anti–IL-5 therapies appear to be effective not only during the acute phase of eosinophilic inflammation but also in later, potentially fibrotic stages of the disease, likely due to the persistence of eosinophilic infiltration even in these phases. Their mechanism of action is particularly relevant, as IL-5 plays a central role in eosinophil recruitment, activation, and survival. In turn, eosinophils release profibrotic mediators such as IL-13, MBP, and eosinophil cationic protein (ECP), which contribute to tissue remodeling and fibrosis (48). Kisling et al. described a case of a woman treated with mepolizumab (300 mg every four weeks) in combination with OCS to achieve disease control. The patient had experienced multiple relapses, largely related to repeated discontinuation of corticosteroid therapy due to adverse effects, including insomnia and anxiety (47). The efficacy of mepolizumab in the management of CEP relapses has been demonstrated both in combination with OCS and as monotherapy (49, 50). In the report by Ciuffreda et al., treatment with mepolizumab 100 mg resulted in rapid clinical and radiological improvement in a patient who relapsed after discontinuation of OCS following eight years of therapy (49). Similarly, Brenard et al. evaluated mepolizumab at doses of 100 and 300 mg and found no significant differences in efficacy between the two regimens; both were associated with a reduction in relapses, decreased OCS requirements, a decline in eosinophil counts, and complete resolution on computed tomography. However, the study was limited by the small sample size but the strength of a relatively homogeneous population in terms of diagnosis, baseline characteristics, and follow-up (45).

Asano et al. defined refractory CEP as a condition requiring maintenance OCS therapy due to repeated relapses (at least two), with an estimated prevalence of approximately 20% among CEP patients (51). In reported case series, the use of biologics in combination with OCS for refractory CEP demonstrated a favorable safety profile and effective disease control without relapse. Improvements in lung function were also observed in patients treated with mepolizumab and benralizumab. This finding is clinically relevant, as functional deterioration occurs in approximately 10% of patients and may be severe in eosinophilic pneumonias. CEP is frequently associated with asthma and may predispose patients to progressive pulmonary fibrosis. This process is thought to be mediated by eosinophil-derived interferon-γ, which promotes collagen deposition and fibrotic remodeling of lung tissue, independently of lymphocytic proliferation and classical pathways of tissue remodeling (3, 52, 53). Notably, this progression appears more common in long-standing disease, as reported in a retrospective analysis by Baqir et al. (54). Once the disease evolves into progressive pulmonary fibrosis—defined by functional decline (e.g., >5% reduction in forced vital capacity), clinical worsening, or radiological progression (55)—clinical management becomes significantly more challenging, both diagnostically and therapeutically, potentially posing an additional therapeutic challenge. In this context, the introduction of antifibrotic therapy may become necessary, while the associated clinical and functional deterioration may further complicate overall disease management.

From a functional perspective, Durieu et al. (56) first demonstrated that the development of an obstructive ventilatory defect is common during the course of CEP and is often associated with elevated eosinophil levels in BALF, even in the absence of overt clinical or radiological abnormalities (3). Moreover, recurrent relapses further contribute to the progressive impairment of lung function. Asano et al. showed that patients with CEP and concomitant asthma at diagnosis are more likely to develop persistent pulmonary function impairment over time. This consideration has important therapeutic implications, as gradual tapering of OCS may potentially worsen respiratory function; consequently, patients are often exposed to prolonged corticosteroid treatment in order to prevent deterioration (51).

Given that long-term OCS use is frequently associated with significant treatment-related comorbidities, Tashiro et al. evaluated the effects of anti–IL-5 therapies across four patient subgroups stratified by comorbidity burden. In patients with diabetes mellitus, benralizumab was associated with the absence of relapses, improvement in lung function, rapid reduction in eosinophil counts within four weeks, and complete discontinuation of OCS therapy, whereas mepolizumab treatment resulted in sustained clinical stability and significant eosinophil reduction. In patients without comorbidities, OCS doses were reduced, although discontinuation was not always achieved. In a subgroup with cardiovascular comorbidities (hypertension, hyperlipidemia, and heart failure), mepolizumab was associated with clinical stability, absence of relapses, reduction in eosinophil counts, and successful OCS discontinuation (57). These findings suggest that anti–IL-5 therapies may represent a valuable steroid-sparing strategy, particularly in patients at high risk of corticosteroid-related complications.

Reslizumab appears to demonstrate efficacy comparable to mepolizumab; however, current evidence remains limited, and its role as a first-line biologic agent has not yet been established. Nevertheless, in patients with immediate hypersensitivity reactions to mepolizumab, reslizumab may represent a safe and effective alternative.

This review has several limitations, primarily related to the quality and heterogeneity of the available evidence. All included studies were observational or consisted of case reports and small case series; therefore, the results should be interpreted with caution. The lack of randomized controlled trials and standardized outcome measures limits the generalizability of these findings. Furthermore, variability in treatment regimens, follow-up duration, and patient selection may have influenced the reported outcomes. Future well-designed prospective studies, including randomized controlled trials, are needed to better define the role of anti–IL-5 therapies in CEP, particularly with regard to long-term efficacy, optimal dosing strategies, and their impact on quality of life and disease progression.

## Conclusion

Over the past two decades, anti–IL-5 therapies have emerged as highly effective and well-tolerated treatments across a broad spectrum of eosinophilic disorders, including asthma, chronic rhinosinusitis with nasal polyps (CRSwNP), hypereosinophilic syndrome (HES), and EGPA. Their benefit extends beyond a mere steroid-sparing effect, as they directly target IL-5, a central driver of eosinophil differentiation, activation, and tissue recruitment, thereby interfering with a key pathogenic pathway shared across eosinophilic diseases.

In contrast, their role in CEP remains insufficiently defined, largely due to the absence of robust prospective data. This gap is particularly striking given the consistent signals of efficacy reported in the available literature. To date, evidence is almost exclusively derived from case reports and small case series, which not only limits the strength of the conclusions but also reflects a persistent under-recognition of the need for dedicated clinical trials in this setting. Critical aspects—including optimal dosing, treatment duration, dosing intervals, and cost-effectiveness—remain largely empirical, highlighting the lack of a standardized therapeutic framework for biologic use in CEP.

Importantly, most published experiences have focused on relapsing or refractory disease, with only anecdotal evidence supporting the use of anti–IL-5 therapies as first-line treatment (58). As a result, the positioning of biologics within the treatment algorithm is still largely arbitrary and driven by clinical necessity rather than evidence-based strategies. This reactive approach may delay the introduction of potentially disease-modifying therapies, thereby prolonging corticosteroid exposure and increasing the risk of cumulative toxicity. A paradigm shift toward earlier intervention could be justified, but this hypothesis urgently requires validation in controlled studies.

Differences in dosing strategies and treatment duration between mepolizumab and benralizumab likely reflect their distinct mechanisms of action. While mepolizumab neutralizes circulating IL-5, benralizumab induces near-complete eosinophil depletion through IL-5 receptor α–mediated antibody-dependent cytotoxicity. This more profound and sustained eosinophil suppression may explain observations suggesting that benralizumab can be discontinued after achieving remission. Indeed, Yakawa proposed that treatment withdrawal may be feasible following clinical, hematological, and radiological resolution, while Izumo reported a striking case in which a single dose of benralizumab was sufficient to control disease relapse (59). Such findings challenge the conventional paradigm of chronic biologic administration and raise the possibility that, at least in selected patients, intermittent or even single-dose strategies may be sufficient.

Similarly, the optimal dosing interval remains undefined. Emerging evidence suggests that extended intervals beyond those established for asthma may be adequate to maintain disease control in CEP (60, 61). If confirmed, this would have substantial implications for treatment burden, healthcare costs, and long-term adherence. However, these observations are based on limited data and should be interpreted with caution until validated in larger cohorts.

At present, the selection of biologic therapy in CEP remains highly individualized and should take into account disease severity, relapse frequency, cumulative corticosteroid toxicity, baseline blood eosinophil counts, coexisting eosinophilic diseases, route and frequency of administration, patient preferences, and local drug availability. In the absence of comparative effectiveness studies, the choice between anti-IL-5 and anti-IL-5 receptor therapies should be guided primarily by the patient’s clinical phenotype and the goal of achieving sustained disease remission while minimizing long-term corticosteroid exposure.

Taken together, these considerations underscore a critical unmet need for high-quality studies to define the role of anti–IL-5 therapies in CEP. Current evidence, although limited, consistently points toward a significant therapeutic potential that is likely underutilized.

Although anti-IL-5 and anti-IL-5 receptor therapies have emerged as promising steroid-sparing strategies for patients with relapsing or corticosteroid-dependent CEP, current evidence remains limited to observational studies and case reports. Consequently, well-designed prospective studies are needed to better define their place in therapy, optimize treatment strategies, identify predictors of response, and establish which patients are most likely to derive long-term benefit from these biologic agents.

Future prospective studies are needed to establish the role of biologic therapy in chronic eosinophilic pneumonia and should adopt standardized efficacy endpoints to enable meaningful comparisons across studies. A proposed trial framework would enroll patients with relapsing or corticosteroid-dependent CEP and evaluate biologic therapy against standard corticosteroid treatment or a predefined steroid-tapering strategy. The primary endpoint should prioritize the corticosteroid-sparing effect, assessed as the proportion of patients achieving complete oral corticosteroid withdrawal or a sustained reduction of ≥50% in the cumulative oral corticosteroid dose while maintaining clinical remission. Key secondary endpoints should include relapse-free survival, time to first relapse, improvement in respiratory symptoms and quality-of-life scores, normalization of peripheral blood and bronchoalveolar lavage eosinophil counts, and standardized radiological outcomes based on quantitative high-resolution computed tomography (HRCT) assessment or complete radiological resolution of pulmonary infiltrates. Additional exploratory endpoints should incorporate pulmonary function parameters, cumulative corticosteroid-related adverse events, healthcare utilization, and patient-reported outcome measures. The adoption of uniform inclusion criteria and standardized outcome measures would facilitate comparisons between biologic agents and accelerate the development of evidence-based treatment recommendations for CEP.

Until such data are available, the use of anti–IL-5 therapies in CEP will remain largely guided by clinical judgment, despite mounting evidence suggesting that a more proactive and structured approach may be warranted.
